# Terahertz circular Airy vortex beams

**DOI:** 10.1038/s41598-017-04373-6

**Published:** 2017-06-20

**Authors:** Changming Liu, Jinsong Liu, Liting Niu, Xuli Wei, Kejia Wang, Zhengang Yang

**Affiliations:** Wuhan National Laboratory for Optoelectronics, Huazhong University of Science and Technology, Wuhan, 430074 Hubei China

## Abstract

Vortex beams have received considerable research interests both in optical and millimeter-wave domain since its potential to be utilized in the wireless communications and novel imaging systems. Many well-known optical beams have been demonstrated to carry orbital angular momentum (OAM), such as Laguerre-Gaussian beams and high-order Bessel beams. Recently, the radially symmetric Airy beams that exhibit an abruptly autofocusing feature are also demonstrated to be capable of carrying OAM in the optical domain. However, due to the lack of efficient devices to manipulate terahertz (THz) beams, it could be a challenge to demonstrate the radially symmetric Airy beams in the THz domain. Here we demonstrate the THz circular Airy vortex beams (CAVBs) with a 0.3-THz continuous wave through 3D printing technology. Assisted by the rapidly 3D-printed phase plates, individual OAM states with topological charge *l* ranging from *l* = 0 to *l* = 3 and a multiplexed OAM state are successfully imposed into the radially symmetric Airy beams. We both numerically and experimentally investigate the propagation dynamics of the generated THz CAVBs, and the simulations agree well with the observations.

## Introduction

Since 1992 Allen *et al*. firstly recognized that OAM is a fundamental property of electromagnetic waves^1^, OAM has seen a lot of great progress in information communication^2, 3^, particle manipulation^4^, quantum optics^5^ and other fields^6–8^. Typical examples including Laguerre-Gaussian beams^1^ and high-order Bessel beams^9^ are able to carry OAM, which are characterized by azimuthally dependent phase *e* ^*ilφ*^, here *φ* is the transverse azimuthal coordinate and *l* is called the OAM quantum number or topological charge number. OAM-carrying beams with different topological charge number are mutually orthogonal to each other and the topological charge *l*, in principle, can take arbitrary integer value in the real space. Thus, these fascinating features make the OAM states very promising for increasing the transmission capacity and spectrum efficiency in the present communication systems. In addition, the OAM-carrying beams exhibit a donut shape intensity with zero amplitude on the optical axis, which could be used in novel imaging systems^10, 11^.

Although most studies of the OAM concentrate in the optical domain, it is still of great interest to explore the potential applications of the OAM in the millimeter-wave and THz domain. Considering the fact that THz can penetrate many opaque materials and has a relatively high frequency compared with the present RF communication band, the combination of OAM and THz wave may further enrich the THz applications^12–18^ in the imaging and communication fields. Recently, radially symmetric Airy beams, which can not only carry the OAM but also exhibit an autofocusing feature, have been demonstrated in the optical domain^19–22^. In these experiments, the implementations of the required complex phase patterns are mostly based on dynamic light modulators, such as commercial spatial light modulators (SLMs) and digital micro-mirror devices. Unfortunately, such dynamic light modulators do not operate in the THz regime because of the lack of materials with the desired THz modulation^23^. Thus, it could be a challenge to demonstrate the radially symmetric Airy beams in the THz domain. With our previous work^24–27^, we believe that the 3D printing technology may offer a suitable way to achieve the goal of efficiently manipulating the THz wave with complex phase patterns.

In this paper, we demonstrate the THz circular Airy vortex beams (CAVBs) with a 0.3-THz continuous wave, which could be defined as the circular Airy beam carrying OAM. Several diffractive phase plates are designed and 3D-printed to form the system for generating the THz CAVBs. In order to verify the OAM state of the generated THz CAVBs with individual OAM state, an interference experiment of the THz CAVBs and the coherent reference Gaussian beam is carried out, and the experimental results unambiguously show that the THz CAVBs with individual OAM states ranging from *l* = 0 to *l* = 3 are successfully generated. Additionally, the propagation dynamics of the generated THz CAVBs with individual OAM state are both numerically and experimentally investigated. Our observations are consistent with the simulation results. Moreover, an effective measuring method based on geometrical transformation is used to measure the OAM content of the multiplexed THz CAVB. The measuring results clearly exhibit that the generated multiplexed THz CAVB carries two OAM states of *l* = 1 and *l* = 3. In general, the THz CAVBs are generated and possess an autofocusing feature, which may enable applications in novel THz imaging system and THz communication links.

## Results

### Concepts for generating the THz CAVBs

The 1D and 2D finite-energy Airy beams have analytical expressions in the Fourier space^28, 29^, which implies that these beams can be firstly generated in the Fourier space and then Fourier transformed back into the real space by a single lens. Such a method can also be implemented in the generation of circular Airy beams (CABs), i.e., radially symmetric Airy beams. Due to great efforts devoted in the study of abruptly autofocusing beams, the CABs are experimentally demonstrated in the optical regime^30, 31^. Based on the Fourier transform (FT) method described above, the usual generation system of the CABs consists of two components, one of which imprints the required phase pattern and the other performs a FT. The required phase pattern is composed of two parts, namely a radial cubic phase and an axicon phase. With the purpose of imposing the OAM into the CABs to produce the CAVBs (CABs carrying OAM), an azimuthally dependent vortex phase is added to the original phase pattern. As a result, the desired phase pattern for generating CAVBs has the expression of

1 $$\varphi (r,\phi )=\alpha {r}^{3}+\beta r+2{\boldsymbol{\pi }}l\phi ,$$

where *α* and *β* are scaling factors, *l* denotes the topological charge number and (*r*, *φ*) are the polar coordinates. Specifically, CABs can be treated as a special case of the CAVBs with *l* = 0. Inspired by the ideal of integrating the FT lens into the front and the back focal planes, the usual system with a 2 *f* length can be modified into a compact one, here *f* is the focal length of the FT lens. As a result, the modified system still contains two components, but the total system length is reduced to1*f*. Hence, the phase profiles of these two components are in the form of

2 $${\varphi }_{1}(r,\phi )=\alpha {r}^{3}+\beta r+2{\boldsymbol{\pi }}l\phi -{k}_{0}{r}^{2}/(2f),\,{\varphi }_{2}(r,\phi )=-{k}_{0}{r}^{2}/(2f),$$

where *k*_0_ = *λ*/2π denotes the wavenumber, *λ* is the wavelength. As we have mentioned the lack of practical devices to manipulate THz wave, imprinting the required phase pattern becomes the barrier in our work. With our previous works in the manipulation of THz wave, we believe that 3D printing technology can be the suitable candidate to imprint such required complex phase patterns. In consideration of the equivalency between phase shift and optical path, when the light penetrates a uniform material with thickness of *h*, its phase shift can be calculated as ΔΨ = 2*π*(*n−*1)*h*/*λ*, where *n* is the refractive index of the material. In addition, considering the size and the material absorption of the refractive components, the phase terms in Equation (2) are wrapped modulo 2π. Finally, our desired phase plates for generating THz CAVBs have the height profiles of

3 $${h}_{1}(r,\phi )=\frac{\lambda }{2{\rm{\pi }}(n-1)}{{\rm{mod}}}_{2{\rm{\pi }}}(\alpha {r}^{3}+\beta r+2{\boldsymbol{\pi }}l\phi -\frac{{k}_{0}{r}^{2}}{2f})+{h}_{0},\,{h}_{2}(r,\phi )=\frac{\lambda }{2{\rm{\pi }}(n-1)}{{\rm{mod}}}_{2{\rm{\pi }}}(-\frac{{k}_{0}{r}^{2}}{2f})+{h}_{0}.$$

A base with a height of *h*_0_ = 2 mm is added to these phase plates to provide a solid basement.

One may notice *h*_1_(*r*, *φ*) only contains a single OAM phase term 2π*lφ*, which can eventually produce the single corresponding OAM state. In order to generate a multiplexed OAM state with a single phase pattern, an Adaptive-Additive algorithm^32^ is applied to design the multiplexed OAM phase term, which can then replace the single OAM phase term 2π*lφ* in Equation (3). Here we intend to produce the multiplexed OAM state containing two power equalized OAM states, namely *l* = 1 and *l* = 3.

### Experimental setup

**Figure 1:**
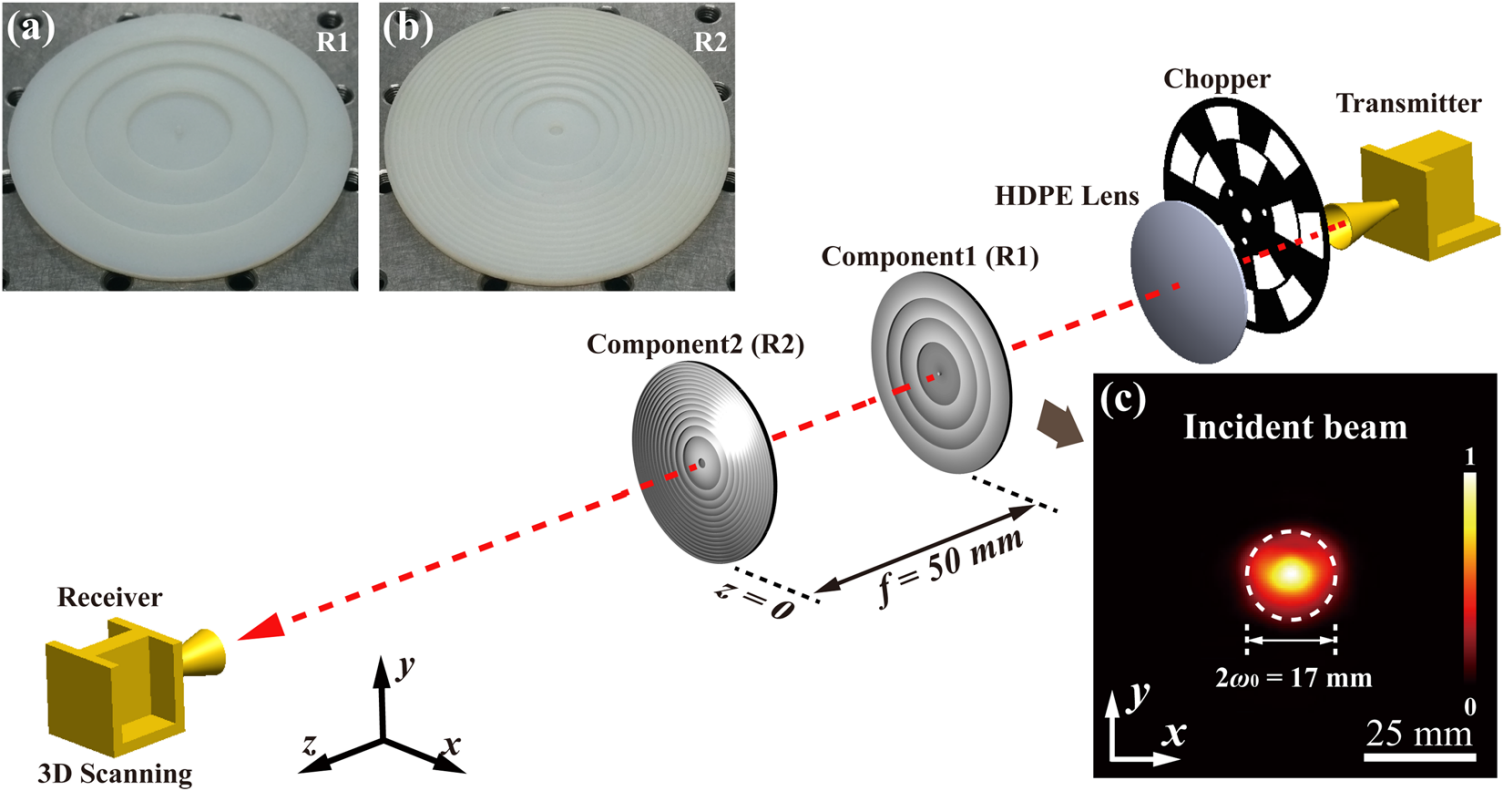
Schematic diagram of the experimental setup for generating THz CAVBs. Transmitter: A 0.3-THz radiation source coupled with a diagonal horn antenna. Chopper: Optical chopper. HDPE lens: High-density polyethylene lens. Receiver: Schotkky diode coupled with a diagonal horn antenna. Component1 (R1): the first phase plate; Component2 (R2): the second phase plate. (**a**) The 3D-printed R1 used in the experiment. (**b**) The 3D-printed R2. (**c**) The *xy* normalized intensity profile of the collimated Gaussian beam at the front plane of R1, showing a diameter of 17 mm (measured at FWHM, full width at half maximum).

The schematic diagram of the experimental setup for generating THz CAVBs is illustrated in Fig. 1. A Gunn diode (Spacek Lab Inc.) driving multiplier-chain (Virginia Diodes Inc.) is utilized as the THz source, which delivers 0.3-THz continuous wave (CW) with an output power of 0.3 mW. The CW THz wave is then emitted into free space through a diagonal horn antenna (WR-3.4, Virginia Diodes Inc.) and perpendicular to the underlying optical table. A high-density polyethylene (HDPE) lens is used to collimate the emitted THz wave. The collimated THz wave is then directed onto the generation system of THz CAVBs, which contains two phase plates, namely Component1 (R1) and Component2 (R2). A Schottky-diode (Virginia Diodes Inc.) in combination with an identical diagonal horn antenna is used as the THz receiver. A lock-in amplifier (SR830, Stanford Research Systems) in cooperation with an optical chopper (in front of the transmitter) is exploited to detect the induced photocurrent in the receiver. To obtain the arbitrary intensity profiles of the THz beams, the receiver is mounted on a motorized three-axis translation stage with a detection volume of 90 × 90 × 300 mm. Considering the trade-off between the image quality and time-consuming, the motor step is carefully set to obtain a spatial resolution of 0.5 mm. In this work, the captured images in the *xy*-plane and *xz*-plane have a pixel size of 181 × 181 and 181 × 301, respectively.

**Figure 2:**
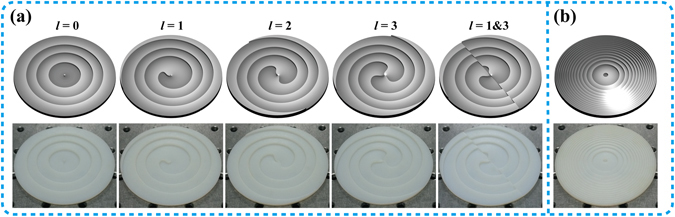
The height profiles of the diffractive phase plates. The CAD models (top row) and the photos (bottom row) of (**a**) The first phase plates (R1) and (**b**) The second phase plate (R2). The parameter *α* = 8.7266 × 10^−4^, *β* = 9.5155. The diameter and the maximum height of these plates are 76.2 mm and 3.53 mm, respectively.

As illustrated in Fig. 1, R1 is the first phase plate of the generation system. A diffractive phase plate with the topological charge *l* = 0 is taking as an example. R2 indicates the second phase plate of the system. Figure 1(a) and 1(b) correspond to the photos of R1 and R2, respectively. Figure 1(c) illustrates the intensity profile of the collimated THz wave at the front plane of R1, which shows a Gaussian-shaped intensity profile with a diameter 2*ω*_0_ = 17 mm (measured at FWHM, full width at half maximum). In order to produce different THz CAVBs, we vary the parameter *l* in Equation (3). Therefore, six phase plates are designed and 3D printed, as depicted in Fig. 2. The top row denotes the CAD models of the phase plates and the bottom row is the corresponding 3D-printed ones. Figure 2(a) and 2(b) refer to R1 and R2, respectively. As we have described above, R1 imprints the required phase pattern into the incident THz Gaussian beam while introducing an additional phase aberration to perform the desired FT. When the FT of the imprinted beam fulfills at the plane of R2, the transformed beam still involves a residual phase aberration. Thus, R2 is implemented to correct the residual phase aberration. With such a system of R1 and R2, the generation of the THz CAVBs would occur right behind R2.

### Generation of CAVBs with individual OAM states ranging from *l* = 0 to *l* = 3

**Figure 3:**
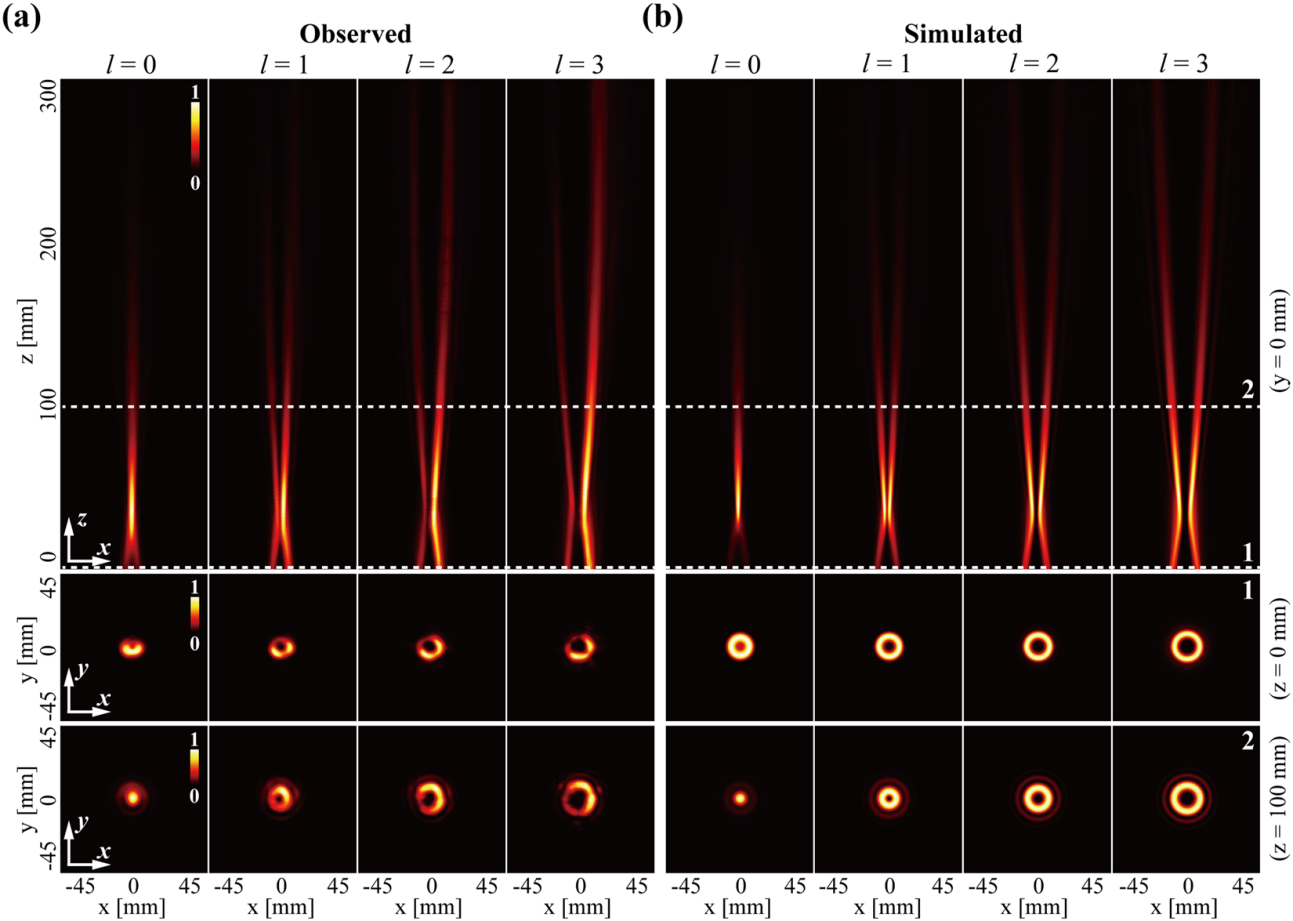
The propagation dynamics of the generated THz CAVBs with individual OAM states ranging from *l* = 0 to *l* = 3. The normalized intensity profiles of (**a**) the experimental results and (**b**) the simulated results for generating THz CAVBs. The first row: intensity profiles in the *xz*-plane (*y* = 0 mm); the second row: intensity profiles in the *xy*-plane (*z* = 0 mm); the third row: intensity profiles in the *xy*-plane (*y* = 100 mm).

With the former four phase plates in Fig. 2(a) and the plate in Fig. 2(b), the THz CAVBs with individual OAM states ranging from *l* = 0 to *l* = 3 could be generated by the system configuration depicted in Fig. 1. For convenience, we define the intersection point of the back plane of R2 and the beam axis as the origin, namely *z* = 0 mm, the other two dimensions are in the similar cases. Therefore, the back plane of R2 perpendicular to the *z*-axis can be called *z* = 0 mm plane and its center locate at *x* = 0 mm, *y* = 0 mm. Assisted by the 3D scanner described above, the 3D intensity distribution of the generated beams could be easily obtained. Hence we can investigate the propagation dynamics of the generated THz CAVBs. We take the advantage of the circular symmetry of the THz CAVBs and first experimentally observe the normalized intensity profiles of the THz CAVBs with the charge *l* ranging from *l* = 0 to *l* = 3 in the *xz*-plane (*y* = 0 mm), as depicted in the first row of Fig. 3(a). In accordance with the prediction, the observed THz CAVBs show an autofocusing feature and contain a typical intensity distribution of the OAM-carrying beams. At the plane of *z* = 0 mm, all these beams exhibit ring-shaped intensity profiles, showing in the second row of Fig. 3(a). The major ring diameters of the CAVBs are gradually reduced during a certain propagation distance, which could lead the beam intensity to considerably increase. After the reducing process, the special THz CAVBs with *l* = 0 forms a solid beam and maintains its shape for more than 100 mm. However, the other three beams with *l* = 1 to *l* = 3 begin to diverge and exhibit a hollow-core intensity distribution along the propagation direction. The third row of Fig. 3(a) illustrates the intensity distributions in the *z* = 100 mm plane, corresponding to the position of the dash line marked as 2 in the first row. The hollow core size is proportional to the absolute value of *l*, which coincides the varying situation of the Laguerre-Gaussian beams and high-order Bessel beams. Moreover, numerical simulations based on the angular spectrum method is implemented, as depicted in Fig. 3(b). Comparing Fig. 3(b) and 3(a), one can easily find out that the numerical simulations agree well with the observations.

**Figure 4:**
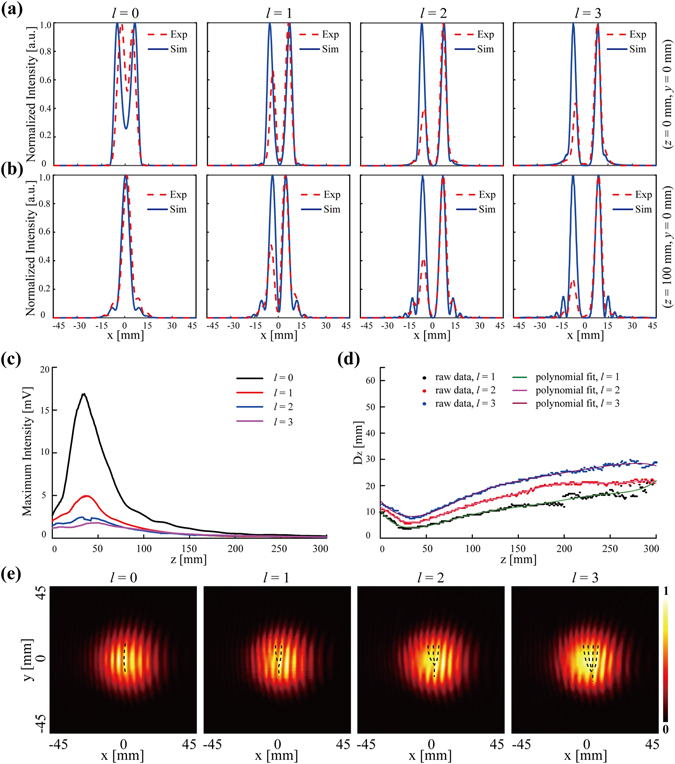
Detailed investigations for generating the THz CAVBs with individual OAM states ranging from *l* = 0 to *l* = 3. (**a**) The 1D normalized intensity curves locates at *z* = 0 mm, *y* = 0 mm. (**b**) The 1D normalized intensity curves locates at *z* = 100 mm, *y* = 0 mm. (**c**) The maximum intensity distribution of THz CAVBs with *l* ranging from *l* = 0 to *l* = 3 along the propagation direction (*z*-axis). (**d**) The ring diameter of the THz CAVBs with *l* ranging from *l* = 1 to *l* = 3 along *z*-axis. (**e**) The interference patterns of the THz CAVBs and a tilted Gaussian beam. Exp: Experiment, Sim: Simulation.

To further investigate the detailed propagation dynamics of the observed results and the simulated ones, 1D normalized intensity curves located at the dash lines of Fig. 3 are depicted in Fig. 4(a) and 4(b), respectively. In general, the peak positions of the red dash lines are almost in coincidence with the ones of the blue solid lines, which indicates that the observed results (red dash lines) are in precise agreement with the simulated ones (blue solid lines). For the last three figures in Fig. 4(a) and 4(b), the intensity value of the left peak is always lower than the value of the right one, which could be caused by the system misalignment. Furthermore, we experimentally investigate the maximum intensity distribution along the propagation direction in Fig. 4(c) and the variations of hollow core diameter of the THz CAVBs with *l* = 1 to *l* = 3 along *z*-axis in Fig. 4(d). All the four curves in Fig. 4(c) show the intensity enhancement during propagation and their peaks are almost located in a same position. The variation of the main ring diameter of the THz CAVBs, which is defined as D_*z*_, coincides with the autofocusing feature in Fig. 4(d).

Various approaches have proven to be able to unambiguously measure the OAM content of the incident beam^33^. In this work, an interference method based on the interference patterns of OAM carrying beams and a tilted coherent Gaussian beam is adopted to verify the individual OAM state of the generated THz CAVBs^34, 35^. A silicon wafer which is placed behind the HDPE lens serves as a THz beam splitter and a silver mirror is utilized to adjust the tilted angle of the coherent Gaussian beam. By suitably placing the silver mirror and adjusting the tilted angle, we can obtain the interference pattern of THz CAVBs and a tilted Gaussian beam at *z* = 300 mm. With the fork-shaped interference patterns, one can easily derive the topological charge value of the OAM carrying beam in the following two steps. First, we define the sign of *l*, here the positive and negative topological charge correspond the prongs up or down direction, respectively. Second, the absolute value of *l* can be implied from the number of prongs *l*_1_ in the interference pattern, satisfying the equation |*l*| = *l*_1_ − 1. The experimental interference intensity patterns of the generated CAVBs with *l* ranging from 0 to 3 are depicted in Fig. 4(e), and the prongs situation is marked as black dash lines. In accordance with the prediction, the number of the prongs equals to *l* + 1, which indicates that the beams carry the desired OAM states.

### Generation of the CAVB with a multiplexed OAM state

**Figure 5:**
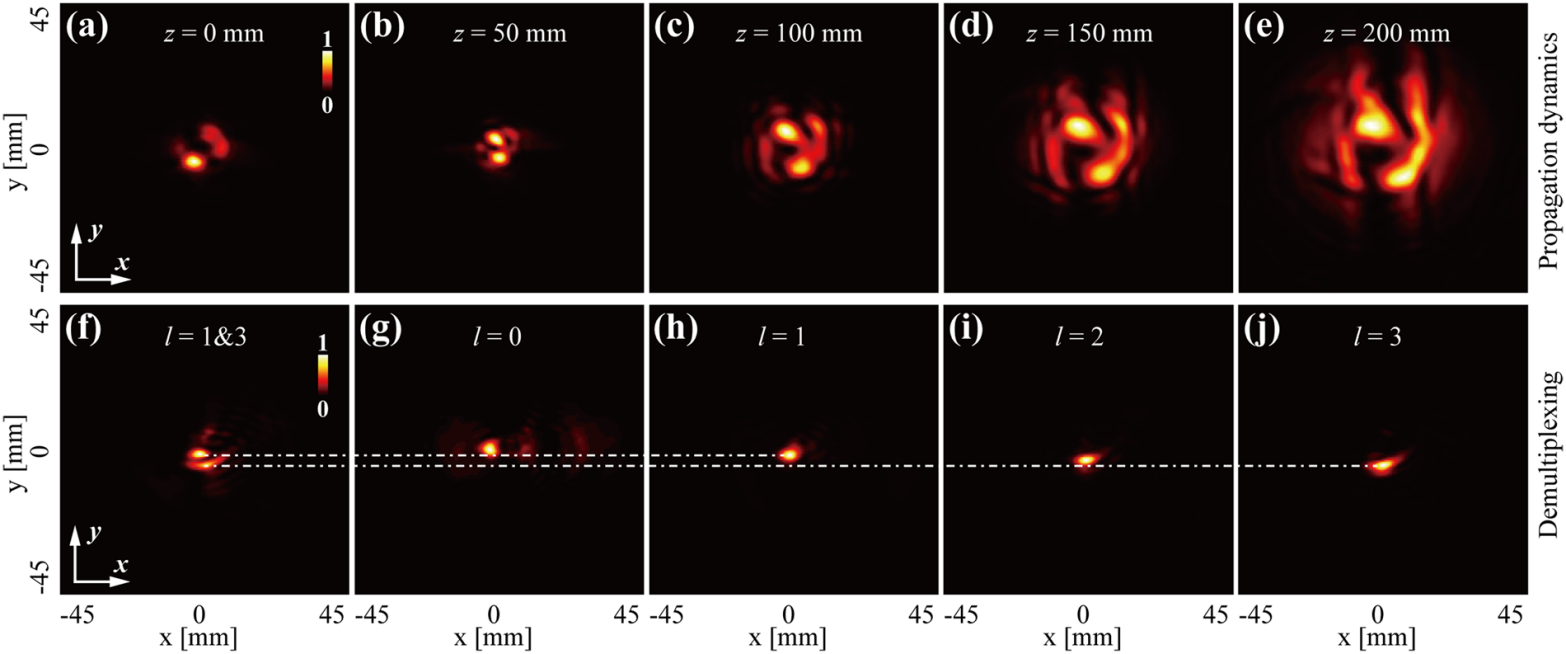
The propagation dynamics and the multiplexing of the multiplexed THz CAVB with *l* = 1&3. (**a**)–(**e**) The *xy* normalized intensity profiles of the multiplexed THz CAVB at the position of *z* = 0 mm, *z* = 50 mm, *z* = 100 mm, *z* = 150 mm and *z* = 200 mm. (**f**) The measured intensity profile of the multiplexed THz CAVB with *l* = 1&3. (**g**)–(**j**) The measured intensity profiles of the THz CAVBs with individual OAM mode ranging from *l* = 0 to *l* = 3.

Generation of the CAVB with a multiplexed OAM state. As described above, we have intended to impose two OAM states of *l* = 1 and *l* = 3 into one CAB. Thus the question arises as to whether the generated THz CAVB carries the desired OAM content. We first investigate the propagation dynamics of the multiplexed THz CAVBs via capturing its *xy* intensity profiles along propagation direction. As depicted in the Fig. 5(a), five normalized intensity profiles with the position of *z* = 0 mm, *z* = 50 mm, *z* = 100 mm, *z* = 150 mm and *z* = 200 mm are captured, showing a tendency that corresponds to the autofocusing feature. In order to verify the OAM content of the multiplexed THz CAVB, a mode sorting method is utilized to measure the OAM content of the input beams in the experiment. Referring to our previous work in the discrimination of OAM states^26^, the measuring system contains a OAM mode transformer and a single lens. Through such a system, different OAM states could be transformed into spots with different positions at the back focal plane of the single lens. The measured intensity profile of the multiplexed THz CAVB is depicted in Fig. 5(f), showing two separated bright spots. For comparison, we further measure the spots of the THz CAVBs with individual OAM states ranging from *l* = 0 to *l* = 3, depicting in Fig. 5(g–j). The positions of the demultiplexed spots in Fig. 5(f) correspond to the ones of Fig. 5(h and j), indicating that the multiplexed THz CAVB carries the desired OAM states of *l* = 1 and *l* = 3.

## Discussion

Our experiments take place in the near-field due to the propagation distance is shorter than the Fraunhofer distance^36^. In the case with sufficient power and suitably designed phase plates, we believe the THz CAVBs could be extended to longer distances in the far field. Although the output power of the present THz source is low and the effect of the atmospheric absorption at 0.3 THz cannot be negligible, the THz CAVBs could still benefit the applications of novel THz imaging systems and short-range THz communications links. For example, the THz CAVB with *l* = 0 shows multiple intriguing features of non-diffraction and autofocusing, which surely can be used to improve the focal depth and imaging capability of the THz imaging systems.

In this paper, we demonstrate the THz CAVBs with a 0.3-THz continuous wave. Six diffractive phase plates are designed and 3D printed to form a compact system, with which the individual OAM states ranging from *l* = 0 to *l* = 3 and a multiplexed OAM state of *l* = 1&3 are generated. We both experimentally and numerically investigate the propagation dynamics of the THz CAVBs, and the experimental results are in good accordance with the simulated ones. Moreover, an interference method is utilized to verify the OAM state of the THz CAVBs with individual OAM, and the interference patterns indicate that the generated beams carry the desired individual OAM states. Based on a geometrical transformation method, the OAM content of the multiplexed THz CAVBs is measured, implying that the multiplexed THz CAVB carries the intended OAM state of *l* = 1&3. In summary, the THz CAVBs have proven to be able to carry OAM states and can abruptly autofocus during propagation, indicating that such beams may have promising applications in the novel THz imaging systems and OAM-based communications links.

## Methods

### Fabrication of the 3D-printed phase plates

Based on Equation (3) above, we obtain the CAD models of the required diffractive phase plates. Then a commercial 3D printer (Objet 30 series, Stratasys Ltd.), having a printing resolution of 600 dpi (42 μm) in the *xy*-plane and 900 dpi (28 μm) along the *z*-axis, is utilized to fabricate these phase plates. The 3D printing material is a rigid opaque material (VeroWhitePlus), whose optical properties are characterized with a Zomega-Z3 THz time-domain spectrometer (THz-TDS) before the fabrication process. At the frequency of 0.3 THz, the refractive index and the absorption coefficient of the printing material are about 1.655 and 1.5 cm^−1^, respectively.

### Numerical simulations of the CAVBs

The angular spectrum method^37^ is exploited to numerically calculate the propagation dynamics of the THz CAVBs. Referring to our system described above, a broad Gaussian beam is directed onto the first diffractive phase plate R1, the electric field of the beam at the back plane of R1 can be

4 $${U}_{1}(r,\phi )=A\,\exp [-{(\frac{r}{{\omega }_{0}})}^{2}]\ast \exp (i{\varphi }_{1}(r,\phi )),$$

where *A* = (*I*_0_)^1/2^, and *I*_0_ is the maximum intensity of the input Gaussian beam. Without loss of generality, material absorption will be neglected. Based on the angular spectrum method, the beam at the front plane of R2 has the expression of

5 $${U}_{2}(u,v)={{\rm{F}}}^{-1}\{{\rm{F}}\,\{{U}_{1}(r,\phi )\}\ast \exp (i\frac{2\pi }{\lambda }f\sqrt{1-{\lambda }^{{\rm{2}}}({f}_{u}^{2}+{f}_{v}^{2})})\}.$$

In Equation (5), (u, *v*) refers to the Cartesian coordinates in the output plane of the generation system, *f*_u_ = *u*/(*λf*) and *f*_v_ = *v*/(*λf*), F and F^−1^ are the Fourier transformation and the inverse Fourier transformation, respectively. Hence the desired THz CAVBs have the filed profiles of

6 $${U}_{3}(x,y)={{\rm{F}}}^{-1}\{{\rm{F}}\,\{{U}_{2}(u,v)\ast \exp (i{\varphi }_{2}(r,\phi ))\}\ast \exp (i\frac{2\pi }{\lambda }Z\sqrt{1-{\lambda }^{2}({f}_{x}^{2}+{f}_{y}^{2})})\},$$

where *Z* is the THz beams’ propagation distance behind the R2, and (*x*, *y*) is the corresponding Cartesian coordinates in the plane perpendicular to the *z*-axis. The parameter *f*_*x*_ and *f*_*y*_ are defined as follows, *f*_*x*_ = *x*/(*λZ*) and *f*_*y*_ = *y*/(*λZ*). Therefore, the free-space 3D electric field of the THz CAVBs could be numerically simulated through the above algorithm step by step.

## References

[1] Allen L, Beijersbergen MW, Spreeuw RJC, Woerdman JP (1992). Orbital angular momentum of light and the transformation of Laguerre-Gaussian laser modes. Phys. Rev. A.

[2] Bozinovic N (2013). Terabit-scale orbital angular momentum mode division multiplexing in fibers. Science.

[3] Gibson G (2004). Free-space information transfer using light beams carrying orbital angular momentum. Opt. Express.

[4] He H, Friese ME, Heckenberg NR, Rubinsztein-Dunlop H (1995). Direct observation of transfer of angular momentum to absorptive particles from a laser beam with a phase singularity. Phys. Rev. Lett..

[5] Vaziri A, Weihs G, Zeilinger A (2002). Experimental two-photon, three-dimensional entanglement for quantum communication. Phys. Rev. Lett..

[6] Mair A, Vaziri A, Weihs G, Zeilinger A (2001). Entanglement of the orbital angular momentum states of photons. Nature.

[7] Barreiro JT, Wei T-C, Kwiat PG (2008). Beating the channel capacity limit for linear photonic superdense coding. Nat. Phys.

[8] Nagali E (2009). Quantum information transfer from spin to orbital angular momentum of photons. Phys. Rev. Lett..

[9] Volke-Sepulveda K, Garcés-Chávez V, Chávez-Cerda S, Arlt J, Dholakia K (2002). Orbital angular momentum of a high-order Bessel light beam. J. Opt. B: Quantum Semiclassical Opt.

[10] Fürhapter S, Jesacher A, Bernet S, Ritsch-Marte M (2005). Spiral phase contrast imaging in microscopy. Opt. Express.

[11] Bernet S, Jesacher A, FŘrhapter S, Maurer C, Ritsch-Marte M (2006). Quantitative imaging of complex samples by spiral phase contrast microscopy. Opt. Express.

[12] Mahmouli FE, Walker SD (2013). 4-Gbps uncompressed video transmission over a 60-GHz orbital angular momentum wireless channel. IEEE Wireless Commun. Lett.

[13] Xie, Z. W. *et al*. Spatial Terahertz Modulator. *Scientific Reports* **3**, doi:10.1038/Srep03347 (2013).

[14] Imai R, Kanda N, Higuchi T, Konishi K, Kuwata-Gonokami M (2014). Generation of broadband terahertz vortex beams. Opt. Lett..

[15] He J (2013). Generation and evolution of the terahertz vortex beam. Opt. Express.

[16] Miyamoto K, Suizu K, Akiba T, Omatsu T (2014). Direct observation of the topological charge of a terahertz vortex beam generated by a Tsurupica spiral phase plate. Appl. Phys. Lett..

[17] Schemmel P, Maccalli S, Pisano G, Maffei B, Ng MWR (2014). Three-dimensional measurements of a millimeter wave orbital angular momentum vortex. Opt. Lett..

[18] Schemmel P, Pisano G, Maffei B (2014). Modular spiral phase plate design for orbital angular momentum generation at millimetre wavelengths. Opt. Express.

[19] Davis JA, Cottrell DM, Sand D (2012). Abruptly autofocusing vortex beams. Opt. Express.

[20] Efremidis NK, Christodoulides DN (2010). Abruptly autofocusing waves. Opt. Lett..

[21] Papazoglou DG, Efremidis NK, Christodoulides DN, Tzortzakis S (2011). Observation of abruptly autofocusing waves. Opt. Lett..

[22] Panagiotopoulos P, Papazoglou DG, Couairon A, Tzortzakis S (2013). Sharply autofocused ring-Airy beams transforming into non-linear intense light bullets. Nat Commun.

[23] Chan WL (2009). A spatial light modulator for terahertz beams. Appl. Phys. Lett..

[24] Zhang Z (2015). Rapid fabrication of terahertz lens via three-dimensional printing technology. Chin. Opt. Lett..

[25] Wei X (2015). Generation of arbitrary order Bessel beams via 3D printed axicons at the terahertz frequency range. Appl. Opt..

[26] Liu C (2016). Discrimination of orbital angular momentum modes of the terahertz vortex beam using a diffractive mode transformer. Opt. Express.

[27] Liu C, Niu L, Wang K, Liu J (2016). 3D-printed diffractive elements induced accelerating terahertz Airy beam. Opt. Express.

[28] Siviloglou GA, Broky J, Dogariu A, Christodoulides DN (2007). Observation of accelerating Airy beams. Phys. Rev. Lett..

[29] Siviloglou GA, Christodoulides DN (2007). Accelerating finite energy Airy beams. Opt. Lett..

[30] Chremmos I (2011). Fourier-space generation of abruptly autofocusing beams and optical bottle beams. Opt. Lett..

[31] Chen B (2015). Propagation of sharply autofocused ring Airy Gaussian vortex beams. Opt. Express.

[32] Lin J, Yuan XC, Tao SH, Burge RE (2005). Collinear superposition of multiple helical beams generated by a single azimuthally modulated phase-only element. Opt. Lett..

[33] Yao AM, Padgett MJ (2011). Orbital angular momentum: origins, behavior and applications. Adv. Opt. Photon..

[34] Bazhenov VY, Soskin M, Vasnetsov M (1992). Screw dislocations in light wavefronts. J. Mod. Opt.

[35] Padgett M, Allen L (2000). Light with a twist in its tail. Contemporary Physics.

[36] Molisch, A. F. *Wireless Communications*. 2nd edn, (Wiley, 2011).

[37] Goodman, J. W. *Introduction to Fourier optics*. (Roberts and Company Publishers, 2005).

